# Synthesis, Molecular Docking and In Vitro Screening of Some Newly Synthesized Triazolopyridine, Pyridotriazine and Pyridine–Pyrazole Hybrid Derivatives

**DOI:** 10.3390/molecules23102548

**Published:** 2018-10-06

**Authors:** Eman M. Flefel, Walaa I. El-Sofany, Mahmoud El-Shahat, Arshi Naqvi, Eman Assirey

**Affiliations:** 1 Department of Chemistry, College of Science, Taibah University, Al-Madinah Al-Monawarah 1343, Saudi Arabia; arshi_84@yahoo.com (A.N.); eman_assirey@hotmail.com (E.A.); 2 Department of Photochemistry, Chemical Industries Research Division, National Research Centre, 33 EL-Bohouth St., Dokki 12622, Giza, Egypt; walaa.elsofany@gmail.com (W.I.E.-S.); mahmoudelshahat@gmail.com (M.E.-S.)

**Keywords:** triazolopyridine, pyridotriazine, pyridine–pyrazole hybrids, antimicrobial activity, antioxidant agent, in silico molecular docking

## Abstract

A series of novel pyridine and fused pyridine derivatives have been prepared starting from 6-(3,4-dimethylphenyl)-2-hydrazinyl-4-(thiophen-2-yl)-pyridine-3-carbonitrile **1** which on treatment with appropriate formic acid, acetic acid/acetic anhydride, benzoyl chloride and/or carbon disulfide afforded the corresponding triazolopyridine derivatives **2**–**5**. Also, treatment of hydrazide **1** with diethyloxalate, chloroacetyl chloride, chloroacetic acid and/or 1,2-dichloroethane yielded the corresponding pyridotriazine derivatives **7**–**10**. Further transformation of compound **1** with a different active methylene group, namely acetyl acetone, diethylmalonate, ethyl cyanoacetate, ethyl benzoylacetate and/or ethyl acetoacetate, produced the pyridine–pyrazole hybrid derivatives **11**–**15**. These newly synthesized compounds (**1**–**15**) were subjected to in silico molecular docking screenings towards GlcN-6-P synthase as the target protein. The results revealed moderate to good binding energies of the ligands on the target protein. All the newly prepared products exhibited antimicrobial and antioxidant activity.

## 1. Introduction

Pyridine derivatives have received great attention because of their presence in various drugs and biologically active molecules [1,2,3,4]. In our previous work, we found that heterocyclic compounds implicate the pyridine nucleus and showed wide promising biological activities such as anti-cancer [5,6,7], anti-oxidant [8,9], anti-microbial [5,9,10] and anti-viral [2] activities. In addition, the literature reports that pyridine derivatives are potent anti-inflammatory [11,12,13,14], Ca^2+^ channel antagonists [15], anti-cancer [16,17,18], anti-plasmodial [19], anti-microbial [20,21], anti-malarial [22] anti-biotic [23], analgesic [24,25], anti-oxidant [26] agents. Also, compounds containing triazolopyridine nucleuses are associated with diverse activities such as anti-fungal [27,28,29,30], insecticidal [30], herbicidal [31], anti-convulsant [32] and anti-bacterial [33,34] activities. Several pyridotriazine analogues were reported to possess various biological activities such as anti-Alzheimer’s [35], anti-fungal [36], anti-microbial [37], anti-thrombotic [38], and hypotensive agents, while also acting as antagonists of serotonin receptors 5-HT2 and 5-HT2a [39,40]. On the other hand, pyrazole hybrid heterocyclic compounds were reported as anti-bacterial [41,42,43,44], anti-inflammatory [42,43,44,45,46], anti-oxidant [46,47], anti-cancer [48,49,50] agents, etc.

In designing a drug candidate, interaction between drug and receptor is mostly understood by employing molecular docking studies. This provides information about the interaction along with predicting the activity and affinity of the targeted drug candidate on the selected receptor [51]. Enzymes involved in the process of biosynthesis of the cell walls of the microbes are considered to be good targets for docking while designing novel compounds such as anti-microbial candidates. In this regard, the enzyme glucosamine-6-phosphate synthase (GlmS, GlcN-6-P synthase, L-glutamine: D-fructose-6P amido-transferase, EC 2.6.1.16) came out to be an attractive target for docking studies in anti-bacterial and anti-fungal drug design [52] as it is involved in the formation of *N*-acetyl Glucosamine (the core amino sugar) which is an important building block of the fungal and the bacterial cell wall [53,54]. Nicotinonitrile-based analogues have been reported as potent anti-bacterial and anti-fungal agents [55,56,57]. Also, some nicotinonitrile derivatives have been reported as inhibitors of GlcN-6-P synthase [57]. Thus, it was thought to be worthwhile to predict drug–receptor interaction employing in silico molecular docking screenings of the synthesized compounds on the selected target i.e., GlcN-6-P synthase.

In line with our previous work [58,59,60,61] and our observations, we propose the modification of new heterocyclic compounds containing a pyridine nucleus in the hope that they could possess good anti-oxidant ability and anti-microbial activity.

## 2. Results and Discussion

### 2.1. Chemistry

**Scheme 1:**
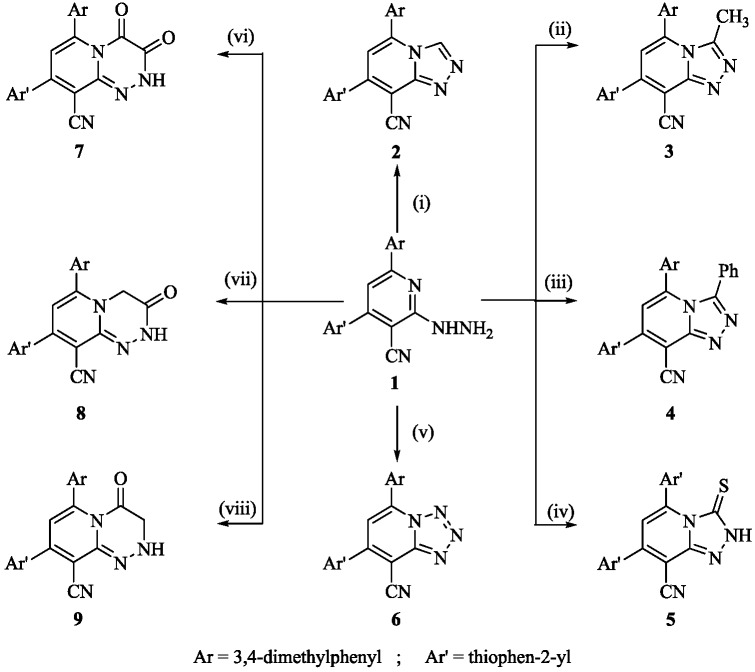
Synthesis of compounds **2**–**9**; reagent and conditions: (i) HCOOH, heat; (ii) CH_3_COOH, (CH_3_CO)_2_O, heat; (iii) PhCOCl, heat; (iv) CS_2_/KOH, heat; (v) NaNO_2_/HCl, rt; (vi) (COOEt)_2_, THF, heat; (vii) ClCH_2_COCl, DMF, heat; (viii) ClCH_2_COOH, DMF, heat.

In the course of our investigation, we have found that 6-(3,4-dimethylphenyl)-2-hydrazinyl-4-(thiophen-2-yl)-pyridine-3-carbonitrile [62] is an excellent building block for the synthesis of a numerous heterocyclic ring systems. Thus, 2-hydrazino-nicotinonitrile **1** reacted with formic acid or acetic acid/acetic anhydride or benzoyl chloride and/or carbon disulfide and afforded the corresponding triazolopyridine derivatives **2**–**5** (Scheme 1). The infrared (IR) spectra of new triazolopyridine derivatives **2**–**5** showed an absorption band characteristic for (CN) at 2213, 2211, 2209 and 2212 cm^−1^, respectively. In addition to the disappearance of the characteristic band for NH, NH_2_ groups of starting 2-hydrazino-nicotinonitrile **1**. The ^1^H nuclear magnetic resonance (NMR) spectrum of compound **2** showed signals at δ = 2.26, 2.28 for two CH_3_ group, 7.23–7.34 & 7.51–7.64 for aromatic–H, 7.92 for pyridine–H and 8.11 for triazole–H. The ^1^H-NMR spectrum of compound **5** showed signals at δ = 2.26, 2.27 for two CH_3_ group, 7.23–7.31 & 7.50–7.62 for aromatic–H, 7.89 for pyridine–H and 8.87 for the NH group, and its mass spectrum showed the molecular ion peak at *m*/*z* (%): 362 (M^+^, 45%) while its base peak was *m*/*z* 304 (M^+^–NCS, 100%) (cf. the Materials and Methods Section).

2-Hydrazino-nicotinonitrile 1 upon diazotization with a solution of sodium nitrite in hydrochloric acid yielded the corresponding tetrazolopyridine derivative 6 (Scheme 1). The IR and ^1^H-NMR spectra of the latter compound revealed the absence of characteristic signals for the NH and NH_2_ groups and its mass spectrum showed molecular ion peak at *m*/*z* (%):331 (M^+^, 37%), 303 (M^+^–N_2_, 100%) (cf. the Materials and Methods Section).

**Scheme 2:**
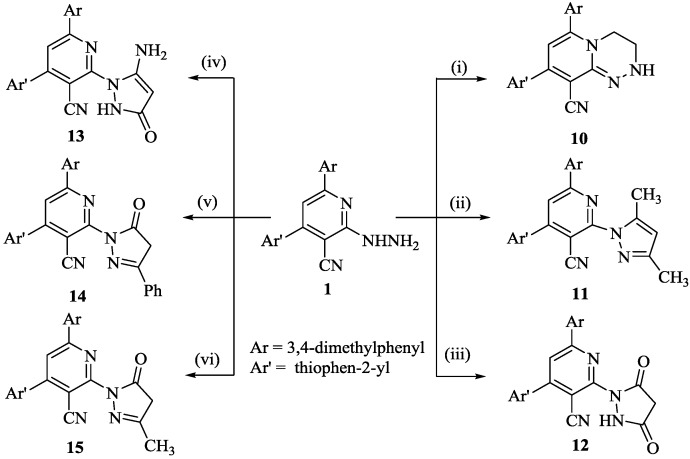
Synthesis of compounds **10**–**15**; reagent and conditions: (i) ClCH_2_CH_2_Cl, DMF, heat; (ii) CH_3_COCH_2_COCH_3_, EtOH, heat; (iii) CH_2_(COOEt)_2_, EtOH, heat; (iv) NCCH_2_CO_2_Et, EtOH, heat; (v) PhCOCH_2_CO_2_Et, EtOH, heat; (vi) 1-CH_3_COCH_2_CO_2_Et, EtOH, heat; 2- EtONa, heat.

On the other hand, treatment of 2-Hydrazino-nicotinonitrile **1** with diethyloxalate, chloroacetyl chloride, chloroacetic acid and/or 1,2-dichloroethane yielded the corresponding pyridotriazine derivatives **7**–**10**, respectively (Scheme 1 and Scheme 2). The IR spectrum of dioxopyridotriazine derivative **7**, for example, showed absorption bands at 3150; 2209; 1713 and 1675 cm^−1^ for (NH); (CN) and two (C=O) groups respectively. Its ^1^H-NMR spectrum showed signals at δ = 2.26, 2.28 for two CH_3_ groups, 7.25–7.34 & 7.49–7.61 for Aromatic–H, 7.90 for pyridine–H, and 9.02 for the NH group; and its mass spectrum showed a molecular ion peak at *m*/*z* (%):374 (M^+^, 22%). Upon spectral measurements the isomeric structures of **8** and **9** were deduced (cf. the Materials and Methods Section). The IR spectrum of pyridotriazine derivative **10** displays absorption bands attributed to (NH) and (CN) and its ^1^H-NMR spectrum showed a signal at δ = 4.51–4.88 for 2CH_2_ groups in addition to two CH_3_ groups, Aromatic–H and pyridine–H (cf. the Materials and Methods Section).

Further transformation of compound **1** with a different active methylene group, namely acetyl acetone, diethylmalonate, ethyl cyanoacetate, ethyl benzoylacetate and/or ethyl acetoacetate, produced the pyridine–pyrazole hybrid derivatives **11**–**15** (Scheme 2). The ^1^H-NMR spectrum of compound **11** displays signals at δ = 2.30, 2.67 for two new CH_3_ groups, 5.96 for pyrazole–H in addition to two CH_3_ group, Aromatic–H and pyridine–H (cf. the Materials and Methods Section). The IR spectrum of compound **12** revealed absorption bands attributed to (NH); (CN) and two (C=O) groups at 3220; 2215; 1729 and 1680 cm^−1^, respectively, and its ^1^H-NMR spectrum showed the signals of CH_2_ and NH protons for the pyrazole ring at δ = 5.13 and 9.08, respectively, in addition to two CH_3_ group, Aromatic–H and pyridine–H (cf. the Materials and Methods Section). The IR spectrum of compound **13** showed bands at 3233; 3117; 2210 and 1685 cm^−1^ for (NH_2_); (NH); (CN) and (C=O) groups, respectively, and its ^1^H-NMR spectrum showed the signals of CH; NH and NH_2_ protons for the pyrazole ring at δ = 6.12; 7.89 and 9.13, respectively, in addition to two CH_3_ group, Aromatic–H and pyridine–H (cf. the Materials and Methods Section). The IR spectra of both **14** and **15** showed bands for (CN) and (C=O) groups at 2217; 1665 and 2215; 1778, respectively. The ^1^H-NMR spectrum showed the signals of CH_2_ for the pyrazole ring at δ = 2.93; 3.10 and 3.71; 3.96, respectively (cf. the Materials and Methods Section).

### 2.2. In Silico Molecular Docking Screenings

**Figure 1:**
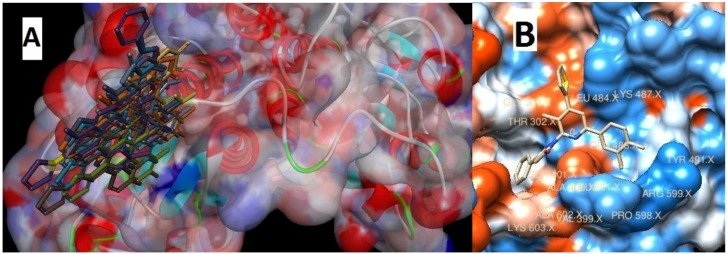
Docking into active site of glucosamine-6-phosphate (GlcN-6-P) synthase (PDB ID: 2VF5). (**A**) Docked poses of compounds **1**–**15** on the GlcN-6-P; (**B**) the interaction between the compound no. 14 and GlcN-6-P.

In silico molecular docking screenings were performed after achieving synthesis and characterization of the compounds. GlcN-6-P synthase was considered as the target receptor. In order to determine the best in silico conformation, comparative and automated docking studies were performed with the newly synthesized drug candidates. All the synthesized compounds (**1**–**15**) were subjected to docking studies on the target protein GlcN-6-P synthase. Figure 1 shows the docked images of these newly synthesized ligand drug candidates on the target protein. The minimum binding energies and estimated inhibition constants of the synthesized compounds are documented in Table 1. The results of in silico studies revealed a moderate to good pattern of affinity and activity of the synthesized compounds on the target protein which is supported by in vitro antimicrobial screenings as the compounds were active against one and/or the other tested microorganisms. Compounds with higher binding energies came out to be more potent than the standard drug taken. Their binding energies ranged from −6.08 to −7.84 kJ/mol with an estimated inhibition constant ranging from 1.79 to 35 micromol.

### 2.3. Pharmacological Screening

#### 2.3.1. Anti-Microbial Activity

All synthesized compounds **1**–**15** were tested for their preliminary in vitro anti-microbial activity against different microorganisms according to reported methods [63] representing fungi (*Aspergillus flavus*; Regional Center for Mycology and Biotechnology (RCMB) 002002 and *Candida albicans*; RCMB 005003 (1) ATCC 10231), Gram-positive bacteria (*Staphylococcus aureus*; RCMB010010 and Bacillus subtilis; RCMB 015 (1) NRRL B-543) and Gram-negative bacteria (*Escherichia coli*; (RCMB 010052) ATCC 25955 and *Salmonella typhimurium*; RCMB 006 (1) ATCC 14028). All microorganisms used were obtained from the RCMB, Al-Azhar University, Egypt. The mean zone of inhibition in mm beyond well diameter (6 mm) produced a range of pathogenic microorganisms. The results are depicted in the following Table 2.

#### 2.3.2. Anti-Oxidant Activity Using 2,2-Diphenyl-1-picrylhydrazyl (DPPH) Scavenging

The antioxidant activity of extract was determined at the RCMB at Al-Azhar University by the 2,2-diphenyl-1-picrylhydrazyl (DPPH) free radical scavenging assay in triplicate and average values were considered. All new prepared pyridine derivatives were screened for anti-oxidant activity according to the reported method [64].

**Figure 2:**
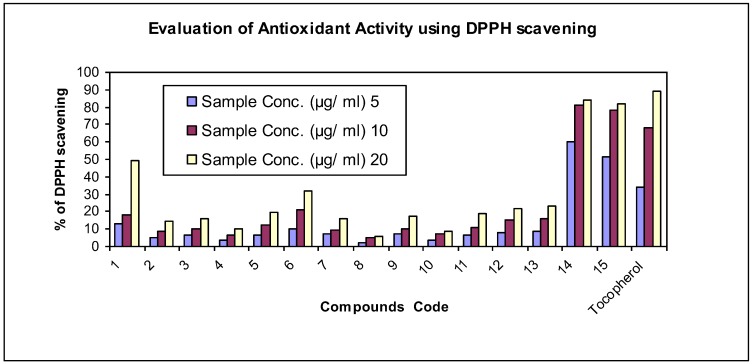
2,2-diphenyl-1-picrylhydrazyl (DPPH) radical scavenging ability of Tocopherol and all synthesized compounds **1**–**15**.

Figure 2 displays DPPH radical scavenging ability of Tocopherol and all synthesized compounds **1**–**15** at different concentrations; compounds **14** and **15** showed excellent anti-oxidant activity at all concentrations due to the introduced 5-oxo-3-Phenyl/Methyl-dihydropyrazole ring. Furthermore, it is apparent that compound **1** which has hydrazide group at the periphery of the molecular chain, has moderate anti-oxidant activity at all concentrations; derivatives **2**–**7**, **9**, **11**–**13** possessed weak anti-oxidant activity at all concentrations while derivatives **8** and **10** showed very weak anti-oxidant activity at all concentrations. All of these results agree with the IC_50_ values shown in Table 3.

#### 2.3.3. Examination of the Structural Activity Relationship (SAR)

The excellent antioxidant activity of the synthesized compounds **14** and **15** depends on the electronic environment and structural skeleton of the molecule. The promising activity of these synthesized motifs is mainly due to the introduction of the 5-oxo-3-Phenyl/Methyl-dihydropyrazole ring into the skeleton.It was observed that replacement on the 3rd position in the dihydropyrazole ring by the phenyl ring or the methyl group dramatically enhances the anti-oxidant activity. Furthermore, it is apparent that compound **1** and compound **6** bearing the hydrazide and tetrazole group, respectively, at the periphery of the molecular chain, have moderate antioxidant activity.

## 3. Materials and Methods

### 3.1. General Information

Melting points were measured using an Electro-Thermal IA 9100 digital melting point apparatus (Büchi, Flawil, Switzerland) and are uncorrected. Infrared spectra were recorded on a Perkin-Elmer 1600 FTIR (Perkin-Elmer, Waltham, MA, USA) discs. NMR spectra were determined on a Jeol-Ex-500 NMR spectrometer (JEOL, Tokyo, Japan) and chemical shifts were expressed as part per million; (δ values, ppm) against TMS as internal reference, National Research Center, Cairo, Egypt. The mass spectra were run at 70 eV with a Finnigan SSQ 7000 spectrometer (Thermo Electron Corporation, Madison, WI, USA) using EI and the values of *m*/*z* are indicated in Dalton. Elemental analyses were performed on a Perkin-Elmer 2400 analyzer (Perkin-Elmer) and were found within the accepted range (± 0.30) of the calculated values. Reaction monitoring and verification of the purity of the compounds was done by TLC on silica gel pre-coated aluminum sheets (type 60 F254, Merck, Darmstadt, Germany). All solvents and chemical reagents were purchased from Aldrich (Munich, Germany). Compound **1** was prepared according to a reported method [62].

### 3.2. Chemistry

#### 3.2.1. 5-(3,4-Dimethylphenyl)-7-(thiophen-2-yl)-[1,2,4]triazolo[4,3-*a*]pyridine-8-carbonitrile (**2**)

A mixture of compound **1** (0.01 mole) and formic acid (20 mL) was refluxed for 20 h. The reaction mixture was filtered off on hot and the separated solid was recrystallized from dioxane to give **2**. Yield 73%, m.p. 162–164 °C. IR (KBr, ν, cm^−1^): 2213 (CN). ^1^H-NMR spectrum (dimethyl sulfoxide (DMSO)-*d*_6_, δ ppm): 2.26 (s, 3H, CH_3_), 2.28 (s, 3H, CH_3_), 7.23–7.34 (m, 3H, Ar–H), 7.51–7.64 (m, 3H, Ar–H), 7.92 (s, 1H, pyridine–H), 8.11(s, 1H, triazole–H). ^13^C-NMR spectrum (CDCl_3_, δ ppm): 19.80, 20.56, 114.16, 115.23, 115.45, 117.41, 120.05, 124.18, 124.29, 126.78, 127.89, 128.58, 128.67, 128.77, 129.35, 131.56, 131.68, 144.38, 159.43; MS, *m*/*z* (%): 330 (M^+^, 52%). Analysis for C_19_H_14_N_4_S (330.41): C, 69.07; H, 4.27; N, 16.96; S, 9.70. Found: C, 68.81; H, 4.06; N, 16. 79; S, 9.41.

#### 3.2.2. 5-(3,4-Dimethylphenyl)-3-methyl-7-(thiophen-2-yl)-[1,2,4]triazolo[4,3-*a*]pyridine-8-carbonitrile (**3**)

A mixture of compound **1** (0.01 mole) and acetic acid/acetic anhydride (30 mL; 2:1) was refluxed for 8 h. The reaction mixture was cooled and poured onto iced-water. The separated solid was filtered off, dried and recrystallized from ethanol to give **3**. Yield 78%, m.p. 199–201 °C. IR (KBr, ν, cm^−1^): 2211 (CN). ^1^H-NMR spectrum (DMSO-*d*_6_, δ ppm): 2.21 (s, 3H, CH_3_), 2.25 (s, 3H, CH_3_), 2.28 (s, 3H, CH_3_), 7.22–7.31 (m, 3H, Ar–H), 7.48–7.60 (m, 3H, Ar–H), 7.88 (s, 1H, pyridine–H). ^13^C-NMR spectrum (CDCl_3_, δ ppm): 17.82, 19.27, 20.83, 114.24, 115.62, 115.81, 117.47, 121.20, 124.51, 124.67, 126.95, 127.87, 128.57, 128.76, 129.66, 129.70, 131.57, 131.81, 144.61, 161.61; MS, *m*/*z* (%): 344 (M^+^, 67%), 329 (M^+^–CH3, 100%). Analysis for C_20_H_16_N_4_S (344.43): C, 69.74; H, 4.68; N, 16.27; S, 9.31. Found: C, 68.47; H, 4.41; N, 16.00; S, 9.05.

#### 3.2.3. 5-(3,4-Dimethylphenyl)-3-phenyl-7-(thiophen-2-yl)-[1,2,4]triazolo[4,3-*a*]pyridine-8-carbonitrile (**4**)

A mixture of compound **1** (0.01 mole), benzoyl chloride (5 mL) and trimethylamine (0.5 mL) was refluxed in ethanol (20 mL) for 8 h. The solvent was removed under reduced pressure and the residue was recrystallized from methanol to give **4**. Yield 59%, m.p. 251–253 °C. IR (KBr, ν, cm^−1^): 2209 (CN). ^1^H-NMR spectrum (DMSO-*d*_6_, δ ppm): 2.26 (s, 3H, CH_3_), 2.28 (s, 3H, CH_3_), 7.22–7.31 (m, 3H, Ar–H), 7.48–7.60 (m, 3H, Ar–H), 7.81–8.48 (m, 6H, 5Ar–H+ pyridine–H).^13^C-NMR spectrum (CDCl_3_, δ ppm): 18.96, 21.15, 113.10, 114.17, 114.38, 116.48, 119.74, 123.23, 126.60, 126.72, 126.97, 127.08, 127.33, 127.52, 127.71, 130.32, 130.49, 133.50, 133.68, 134.11, 134.21, 134.31, 143.39, 151.68, 153.78; MS, *m*/*z* (%): 406 (M^+^, 73%), 329 (M^+^–Ph, 100%). Analysis for C_25_H_18_N_4_S (406.5): C, 73.87; H, 4.46; N, 13.78; S, 7.89. Found: C, 73.61; H, 4.17; N, 13.49; S, 7.59.

#### 3.2.4. 5-(3,4-Dimethylphenyl)-7-(thiophen-2-yl)-3-thioxo-2,3-dihydro-[1,2,4]triazolo[4,3-*a*]pyridine-8-carbonitrile (**5**)

To an aqueous solution of **1** (0.01 mole) in ethanol (20 mL), carbon disulfide (10 mL) was added then the reaction mixture was refluxed in a water-bath for 3 h, cooled, poured onto iced-water and neutralized with 2–3 drops of hydrochloric acid (35%). The precipitate was filtered off, left to dry and recrystallized from methanol to give **5**. Yield 69 %; m.p. 136–138 °C. IR spectrum (KBr, ν, cm^−1^): 3115 (NH); 2212 (CN). ^1^H-NMR (DMSO-*d*_6_, δ ppm): 2.26 (s, 3H, CH_3_), 2.27 (s, 3H, CH_3_), 7.23–7.31 (m, 3H, Ar–H), 7.50–7.62 (m, 3H, Ar–H), 7.89 (s, 1H, pyridine–H), 8.87(s, 1H, NH; D_2_O exchangeable). ^13^C-NMR spectrum (CDCl_3_, δ ppm): 18.81, 21.10, 133.67, 114.74, 114.96, 117.06, 120.32, 123.81, 127.18, 127.29, 127.55, 127.65, 127.90, 128.10, 128.29, 130.90, 131.07, 143.96, 160.70; Ms, *m*/*z* (%): 362 (M^+^, 45), 304 (M^+^–NCS, 100). Analysis for C_19_H_14_N_4_S_2_: C, 62.96; H, 3.89; N, 15.46; S, 17.69. Found: C, 62.68; H, 3.60; N, 15.18; S, 17.41.

#### 3.2.5. 5-(3,4-Dimethylphenyl)-7-(thiophen-2-yl)tetrazolo[1,5-*a*]pyridine-8-carbonitrile (**6**)

To an ice-cold solution of compound **1** (0.01 mole) in hydrochloric acid (35%, 10 mL), a solution of sodium nitrite [prepared by dissolving sodium nitrite (0.01 mole) in water (3 mL)] was added drowsily in an ice-bath. The reaction mixture was allowed to stand overnight at room temperature and then was poured onto water. The formed solid was filtered off; washed with water; dried and recrystallized from ethanol to give **6**. Yield 47%, m.p.175–177 °C. IR (KBr, ν, cm^−1^): 2213 (CN). ^1^H-NMR spectrum (DMSO-*d*_6_, δ ppm): 2.26 (s, 3H, CH_3_), 2.28 (s, 3H, CH_3_), 7.23–7.31 (m, 3H, Ar–H), 7.50–7.62 (m, 3H, Ar–H), 7.89 (s, 1H, pyridine–H). ^13^C-NMR spectrum (CDCl_3_, δ ppm): 18.61, 20.81, 114.81, 115.80, 116.01, 118.00, 121.56, 124.99, 125.18, 127.37, 128.16, 128.77, 129.16, 129.96, 132.29, 136.96, 138.72, 145.07; MS, *m*/*z* (%): 331 (M^+^, 37%), 303 (M^+^–N_2_, 100%). Analysis for C_18_H_13_N_5_S (331.39): C, 65.24; H, 3.95; N, 21.13; S, 9.68. Found C, 64.95; H, 3.67; N, 20.88; S, 9.41.

#### 3.2.6. 6-(3,4-Dimethylphenyl)-3,4-dioxo-8-(thiophen-2-yl)-3,4-dihydro-*2H*-pyrido[2,1-*c*][1,2,4] triazine-9-carbonitrile (**7**)

A mixture of compound **1** (0.01 mole) and diethyl oxalate (0.01 mole) in THF (50 mL) was refluxed for 24 h and then cooled. The solid obtained was filtered off and recrystallized from acetic acid to give **7**. Yield 56%, m.p. 233–235 °C. IR spectrum (KBr, ν, cm^−1^): 3150 (NH); 2209 (CN); 1713 (C=O); 1675 (C=O). ^1^H-NMR (DMSO-*d*_6_, δ ppm): 2.26 (s, 3H, CH_3_), 2.28 (s, 3H, CH_3_), 7.25–7.34 (m, 3H, Ar–H), 7.49–7.61(m, 3H, Ar–H), 7.90 (s, 1H, pyridine–H), 9.02 (s, 1H, NH, D_2_O exchangeable). ^13^C-NMR spectrum (CDCl_3_, δ ppm): 19.33, 21.53, 115.53, 116.52, 116.73, 118.72, 122.28, 125.71, 125.90, 128.08, 128.50, 128.87, 129.49, 129.88, 130.67, 133.01, 137.67, 139.44, 159.68, 162.69; MS, *m*/*z* (%): 374 (M^+^, 22). Analysis for C_20_H_14_N_4_O_2_S: C, 64.16; H, 3.77; N, 14.96; S, 8.56. Found C, 63.88; H, 3.48; N, 14.68; S, 8.29.

#### 3.2.7. 6-(3,4-Dimethylphenyl)-3-oxo-8-(thiophen-2-yl)-3,4-dihydro-*2H*-pyrido[2,1-*c*][1,2,4]triazine-9-carbonitrile (**8**)

To a solution of compound **1** (0.01 mole) in DMF (30 mL), chloroacetyl chloride (0.01 mole) was added dropwise under stirring at room temperature. The reaction mixture was then heated for 12 h and after cooling, poured onto iced-water with vigorous stirring. The precipitate was collected by filtration, washed with water, dried and recrystallized from DMF to give **8**. Yield 47%; m.p. 208–210 °C. IR spectrum (KBr, ν, cm^−1^): 3120 (NH); 2210 (CN); 1675 (C=O). ^1^H-NMR (DMSO-*d*_6_, δ ppm): 2.26 (s, 3H, CH_3_), 2.27 (s, 3H, CH_3_), 4.79 (d, *J* = 9.10; 1H, CH_2_), 4.87 (d, *J* = 9.17, 1H, CH_2_), 7.24–7.32 (m, 3H, Ar–H), 7.48–7.60 (m, 3H, Ar–H), 7.90 (s, 1H, pyridine–H), 8.51 (s, 1H, NH, D_2_O exchangeable). ^13^C-NMR spectrum (CDCl_3_, δ ppm): 18.65, 21.10, 62.15, 113.53, 114.52, 114.73, 116.72, 120.28, 123.71, 123.90, 126.08, 126.50, 126.87, 127.49, 127.88, 128.67, 131.01, 135.67, 137.44, 160.69; Ms, *m*/*z* (%): 360 (M^+^, 37). Analysis for C_20_H_16_N_4_OS: C, 66.65; H, 4.47; N, 15.54; S, 8.90. Found: C, 66.37; H, 4.20; N, 15.25; S, 8.66.

#### 3.2.8. 6-(3,4-Dimethylphenyl)-4-oxo-8-(thiophen-2-yl)-3,4-dihydro-*2H*-pyrido[2,1-*c*][1,2,4]triazine-9-carbonitrile (**9**)

To a solution of compound **1** (0.01 mole) in DMF (30 mL), chloroacetic acid (0.01 mole) was added drop wise under stirring at room temperature. The reaction mixture was then heated for 3 h and after cooling, poured onto iced-water with vigorous stirring. The precipitate was collected by filtration, washed with water, dried and recrystallized from DMF to give **9**. Yield 60%; m.p. 189–191 °C. IR spectrum (KBr, ν, cm^−1^): 3175 (NH); 2211(CN); 1667 (C=O). ^1^H-NMR (DMSO-*d*_6_, δ ppm): 2.26 (s, 3H, CH_3_), 2.28 (s, 3H, CH_3_), 4.17 (d, *J* = 8.92 Hz, 1H, CH_2_), 4.22 (d; *J* = 9.00 Hz; 1H, CH_2_), 7.25–7.34 (m, 3H, Ar–H), 7.49–7.61(m, 3H, Ar–H), 7.90 (s, 1H, pyridine–H), 8.61 (s, 1H, NH, D_2_O exchangeable). ^13^C-NMR spectrum (CDCl_3_, δ ppm): 18.65, 21.10, 48.24, 113.53, 114.52, 114.73, 116.72, 120.28, 123.71, 123.90, 126.08, 126.50, 126.87, 127.49, 127.88, 128.67, 131.01, 135.67, 137.44, 167.48; Ms, *m*/*z* (%): 360 (M^+^, 45). Analysis for C_20_H_16_N_4_OS: C, 66.65; H, 4.47; N, 15.54; S, 8.90. Found: C, 66.40; H, 4.18; N, 15.27; S, 8.64.

#### 3.2.9. 6-(3,4-Dimethylphenyl)-8-(thiophen-2-yl)-3,4-dihydro-*2H*-pyrido[2,1-*c*][1,2,4]triazine-9-carbonitrile (**10**)

A mixture of compound **1** (0.01 mole) and 1,2-dichloroethane (0.01 mole) in DMF (30 mL) was refluxed for 4 h, then poured onto crushed ice. The solid which separated was filtered off and dried then recrystallized from THF to give **10**. Yield 58%, m.p. 260–262 °C. IR (KBr, ν, cm^−1^): 3209 (NH); 2210 (CN). ^1^H-NMR spectrum (DMSO-*d*_6_, δ ppm): 2.25 (s, 3H, CH_3_), 2.28 (s, 3H, CH_3_), 4.51–4.82 (m, 4H, 2CH_2_), 7.20–7.30 (m, 3H, Ar–H), 7.53–7.61 (m, 3H, Ar–H), 7.86 (s, 1H, pyridine–H), 8.67(s, 1H, NH, D_2_O exchangeable). ^13^C-NMR spectrum (CDCl_3_, δ ppm): 18.65, 21.10, 41.24, 43.44, 113.53, 114.52, 114.73, 114.82, 116.72, 120.28, 123.71, 123.90, 126.08, 126.50, 127.49, 127.88, 128.67, 131.01, 135.67,137.44; MS, *m*/*z* (%): 346 (M^+^, 57%). Analysis for C_20_H_18_N_4_S (346.45): C, 69.34; H, 5.24; N, 16.17; S, 9.26. Found: C, 69.09; H, 4.95; N, 15.87; S, 8.98.

#### 3.2.10. 2-(3,5-Dimethyl-*1H*-pyrazol-1-yl)-6-(3,4-dimethylphenyl)-4-(thiophen-2-yl)nicotinonitrile (**11**)

A mixture of compound **1** (0.01 mole) and acetyl acetone (0.02 mole) in ethanol (20 mL) was refluxed for 10 h. The separated solid was filtered off, dried and recrystallized from dioxane to give **11**. Yield 79%, m.p. 224–226 °C. IR (KBr, ν, cm^−1^): 2210 (CN). ^1^H-NMR spectrum (DMSO-*d*_6_, δ ppm): 2.26 (s, 3H, CH_3_), 2.27 (s, 3H, CH_3_), 2.30 (s, 3H, CH_3_), 2.67 (s, 3H, CH_3_), 5.96 (s, 1H, pyrazole–H), 7.22–7.31 (m, 3H, Ar–H), 7.52–7.61 (m, 3H, Ar–H), 7.88 (s, 1H, pyridine–H). ^13^C-NMR spectrum (CDCl_3_, δ ppm): 15.81, 16.89, 18.78, 20.04, 111.81, 113.48, 115.13, 118.93, 121.66, 122.25, 123.97, 124.83, 126.90, 128.99, 130.90, 132.37, 132.77,134.68, 136.16, 137.61, 138.68, 141.39, 142.87; MS, *m*/*z* (%): 384 (M^+^, 41%). Analysis for C_23_H_20_N_4_S (384.5): C, 71.85; H, 5.24; N, 14.57; S, 8.34. Found: C, 71.57; H, 4.97; N, 14.28; S, 8.06.

#### 3.2.11. 6-(3,4-Dimethylphenyl)-2-(3,5-dioxopyrazolidin-1-yl)-4-(thiophen-2-yl)nicotinonitrile (**12**)

A mixture of compound **1** (0.01 mole) and diethylmalonate (0.01 mole) was fused for 1 h then absolute ethanol (20 mL) was added drop wise and reflux continued for additional 5 h. The solid product formed was filtered off and recrystallized from dioxane to give **12**. Yield 70%; m.p. 289–291 °C. IR spectrum (KBr, ν, cm^−1^): 3220 (NH); 2215 (CN); 1729 (C=O); 1680 (C=O). ^1^H-NMR (DMSO-*d*_6_, δ ppm): 2.26 (s, 3H, CH_3_), 2.28 (s, 3H, CH_3_), 5.13 (s, 2H, CH_2_), 7.20–7.28 (m, 3H, Ar–H), 7.47–7.58 (m, 3H, Ar–H), 7.89 (s, 1H, pyridine–H); 9.08 (s, 1H, NH; D_2_O exchangeable). ^13^C-NMR spectrum (CDCl_3_, δ ppm): 19.00, 20.04, 50.08, 111.77, 115.40, 118.78, 122.15, 123.97, 124.72, 125.52, 127.33, 129.43, 132.53, 134.89, 137.46, 140.01, 141.83, 142.91, 145.00, 157.76, 167.95; Ms, *m*/*z* (%): 388 (M^+^, 29). Analysis for C_21_H_16_N_4_O_2_S: C, 64.93; H, 4.15; N, 14.42; S, 8.25. Found: C, 64.66; H, 3.87; N, 14.15; S, 7.96.

#### 3.2.12. 2-(5-Amino-3-oxo-2,3-dihydropyrazol-1-yl)-6-(3,4-dimethylphenyl)-4-(thiophen-2-yl)nicotinonitrile (**13**)

A mixture of compound **1** (0.01 mole) and ethyl cyanoacetate (0.02 mole) was fused for 1 h then absolute ethanol (20 mL) was added drop wise and reflux continued for additional 2 h. The solid product formed was filtered off and recrystallized from dioxane to give **13**. Yield 51%; m.p. 219–221 °C. IR spectrum (KBr, ν, cm^−1^): 3233 (NH_2_); 3117 (NH); 2210 (CN); 1685 (C=O). ^1^H-NMR (DMSO-*d*_6_, δ ppm): 2.26 (s, 3H, CH_3_), 2.28 (s, 3H, CH_3_), 6.12 (s, 1H, pyrazole–H), 6.80 (s, 2H, NH_2_; D_2_O exchangeable), 7.21–7.29 (m, 3H, Ar–H), 7.49–7.57 (m, 3H, Ar–H), 7.89 (s, 1H, pyridine–H); 9.13 (s, 1H, NH; D_2_O exchangeable). ^13^C-NMR spectrum (CDCl_3_, δ ppm): 19.00, 20.04, 94.50, 111.83, 114.72, 118.66, 121.80, 123.93, 124.95, 125.20, 127.33, 129.39, 131.00, 132,80, 134.61, 135.98, 141.45, 143.08, 144.64, 145.97, 169.81; Ms, *m*/*z* (%): 387 (M^+^, 19). Analysis for C_21_H_17_N_5_OS: C, 65.10; H, 4.42; N, 18.08; S, 8.28. Found: C, 64.82; H, 4.17; N, 17.79; S, 8.01.

#### 3.2.13. 6-(3,4-Dimethylphenyl)-2-(5-oxo-3-phenyl-4,5-dihydropyrazol-1-yl)-4-(thiophen-2-yl)nicotinonitrile (**14**)

A mixture of compound **1** (0.01 mole) and ethyl benzoylacetate (0.02 mole) was fused for 1 h then absolute ethanol (20 mL) was added drop wise and reflux continued for additional 4 h. The solid product formed was filtered off and recrystallized from dioxane to give compound **14**. Yield 63%; m.p. 279–281 °C. IR spectrum (KBr, ν, cm^−1^): 2217 (CN); 1665 (C=O). ^1^H-NMR (DMSO-*d*_6_, δ ppm): 2.26 (s, 3H, CH_3_), 2.28 (s, 3H, CH_3_), 2.93 (d, *J* = 8.98 Hz, 1H, CH_2_), 3.10 (d, *J* = 8.96 Hz, 1H, CH_2_), 7.22–7.31 (m, 3H, Ar–H), 7.48–7.60 (m, 3H, Ar–H), 7.81-8.48 (m, 6H, 5Ar–H+ pyridine–H). ^13^C-NMR spectrum (CDCl_3_, δ ppm): 19.43, 20.90, 36.89, 113.91, 117.13, 120.93, 122.88, 123.46, 124.39, 125.10, 126.00, 126.92, 127.07, 129.32, 131.30, 132.54, 133.26, 133.58, 134.75, 136.72, 137.44, 138.87, 139.57, 140.63, 143.07, 145.12,175.60; Ms, *m*/*z* (%): 448 (M^+^, 34), 371 (M^+^–Ph, 100). Analysis for C_27_H_20_N_4_OS: C, 72.30; H, 4.49; N, 12.49; S, 7.15. Found: C, 72.02; H, 4.21; N, 12.20; S, 6.89.

#### 3.2.14. 6-(3,4-Dimethylphenyl)-2-(3-methyl-5-oxo-4,5-dihydropyrazol-1-yl)-4-(thiophen-2-yl)nicotinonitrile (**15**)

To a solution of compound **1** (0.01 mole) and ethyl acetoacetate (0.01 mole) was fused for 1 h then absolute ethanol (20 mL) was added drop wise and reflux continued for additional 2 h. The solid product which formed was refluxed with sodium ethoxide (20 mL) for 0.5 h. The solid precipitate was filtered off and recrystallized from acetic acid to give compound **15**. Yield 51%, m.p. 263–265 °C. IR spectrum (KBr, ν, cm^−1^): 2215(CN), 1678 (C=O). ^1^H-NMR (DMSO-*d*_6_, δ ppm): 2.20 (s, 3H, CH_3_); 2.24 (s, 3H, CH_3_), 2.27 (s, 3H, CH_3_), 3.71 (d, *J* = 8.87 Hz, 1H, CH_2_), 3.96 (d, *J* = 8.86 Hz, 1H, CH_2_), 7.21–7.29 (m, 3H, Ar–H), 7.49–7.57 (m, 3H, Ar-H), 7.89 (s, 1H, pyridine–H). ^13^C-NMR spectrum (CDCl_3_, δ ppm): 18.99, 20.96, 21.65, 45.48, 113.99, 116.72, 120.95, 126.17, 126.84, 127.07, 128.44, 129.89, 131.37, 133.07, 134.38, 136.36, 138.13, 139.60, 141.82, 143.38, 144.82, 175.45; MS, *m*/*z* (%): 386 (M^+^, 46), 371(M^+^–CH_3_, 100). Analysis for C_22_H_18_N_4_OS: C, 68.37; H, 4.69; N, 14.50; S, 8.30. Found: C, 68.10; H, 4.40; N, 14.23; S, 8.03.

### 3.3. In Silico Molecular Docking Screenings

A Lamarckian genetic algorithm contained in AutoDock (version 4.0) was employed for in silico molecular docking studies [65] as it is more reliable, efficient, successful and possesses more degree of freedom for ligands as compared to the Monte Carlo method present in old versions of AutoDock. ChemSketch was used for drawing the ligands and that file format was converted into PDB format using Open Babel [66]. Glucosamine-6-phosphate of GlcN-6-P synthase (PDB ID 2VF5) was obtained from Protein Data Bank (http://www.pdb.org/pdb/home/home.do) with the best accurate active region as solved by experimental crystallographic data [67]. This protein was then optimized by removing all the heteroatoms, rotating all the torsions and adding the C-terminal oxygen. Its energy was minimized using by PRODRG server [68]. Ligand was added with polar hydrogen’s along with assignment of Kollman partial charges. We made and adjusted the grid in *X*, *Y*, *Z*-axis so as to cover the entire active site of protein i.e., the 12 amino acids residues (Cys300, Gly301, Thr 302, Ser 303, Ser 347, Gln 348, Ser 349, Thr 352, Val 399, Ala 400, Ala 602 and Lys 603) by setting the grid box size at 70, 64, and 56 Å for x, y and z, respectively, and the grid center to 30.59, 15.822 and 3.497 for x, y and z, respectively, with grid spacing of 0.375 Å. Standard protocol was undertaken for docking studies. The docking results were interpreted according to the .pdb file. The co-ordinates of the minimum energy run were determined using the rmsd table created in the .dlg file. UCSF Chimera 1.11.2 was used to visualize this ligand protein interaction within region of 6.5 Ǻ.

### 3.4. In Vitro Anti-Microbial Screenings

The Susceptibility Tests were performed according to National Committee for Clinical Laboratory Standards (NCCLS) recommendations (1993). Screening tests regarding the inhibition zone were carried out by the well diffusion method according to Hindler’s method [63]. The inoculum suspension was prepared from colonies grown overnight on an agar plate and inoculated into Mueller–Hinton broth (fungi using malt broth). A sterile swab was immersed in the suspension and used to inoculate Mueller-Hinton agar plates (fungi using malt agar plates). The compounds were dissolved in DMSO with different concentrations (10, 5, 2.5 mg/mL). The inhibition zone was measured around each well after 24 h at 37 °C. Controls using DMSO were adequately done.

### 3.5. DPPH Radical Scavenging Activity

Freshly prepared (0.004% *w*/*v*) methanol solution of DPPH radical was prepared and stored at 10 °C in the dark. A methanol solution of the test compound was prepared. A 40 uL aliquot of the methanol solution was added to 3 ml of DPPH solution. Absorbance measurements were recorded immediately with a ultraviolet (UV)-visible spectrophotometer (Milton Roy, Spectronic 1201). The decrease in absorbance at 515 nm was determined continuously, with data being recorded at 1 min intervals until the absorbance stabilized (16 min). The absorbance of the DPPH radical without anti-oxidant (control) and the reference compound ascorbic acid were also measured. All the determinations were performed in three replicates and averaged. The percentage inhibition (PI) of the DPPH radical was calculated according to the formula:

PI = {(*A_C_* − *A_T_*)/*A_C_*} × 100 (1)

where *A_C_* = Absorbance of the control at *t* = 0 min and *A_T_* = absorbance of the sample + DPPH at *t* = 16 min.

## 4. Conclusions

This study focused on the synthesis of new nicotinonitrile derivatives which were synthesized and characterized using spectral and elemental analyses. All synthesized compounds **1**–**15** were screened for anti-oxidant activity. Compounds **14** and **15** showed excellent anti-oxidant activity at all concentrations due to the introduced 5-oxo-3-Phenyl/Methyl-dihydropyrazole ring. Furthermore, it is apparent that compound **1** which has a hydrazide group at the periphery of the molecular chain has moderate anti-oxidant activity. In silico studies were undertaken to predict the activity and affinity of the synthesized compounds towards the target protein i.e., Glucosamine-6-phosphate synthase and the results were reported to be ranging from −6.08 to −7.84 kJ/mol along with estimation of inhibition constant, Ki, from 1.79–35 micromol. Also, the prepared compounds were tested for their preliminary in vitro anti-microbial activity against different microorganisms. Some of the compounds came up as active against screened bacterial and fungal strains while other were non-reactive. The in silico and in vitro results are consistent with each other as some of the synthesized compounds have demonstrated moderate to good anti-bacterial and anti-fungal activities as compared to the standard reference drugs.

## Figures and Tables

**Table 1:** In silico molecular docking results of the synthesized compounds (**1**–**15**).

Compound Code	Minimum Binding Energy (kJ/mol)	Estimated Inhibition Constant, Ki = uM (micromol)
**1**	−6.08	35 uM
**2**	−7.19	5.39 uM
**3**	−7.34	4.16 uM
**4**	−7.55	2.94 uM
**5**	−6.28	25.04 uM
**6**	−7.51	3.13 uM
**7**	−7.77	2.00 uM
**8**	−7.84	1.79 uM
**9**	−7.29	4.51 uM
**10**	−7.62	2.59 uM
**11**	−6.55	15.69 uM
**12**	−6.15	30.87 uM
**13**	−6.29	24.67 uM
**14**	−7.03	7.00 uM
**15**	−6.10	33.94 uM

**Table 2:** Anti-microbial activities of newly synthesized compounds **1**–**15**.

Sample Code Tested Microorganisms	1	2	3	4	5	6	7	8	9	10	11	12	13	14	15	Control
FUNGI																*Ketoconazole*
*Aspergillus flavus* (RCMB 002002)	NA	NA	NA	7	12	8	9	11	NA	11	10	7	8	7	NA	16
*Candida albicans* RCMB 005003 (1) ATCC 10231	9	NA	NA	NA	12	11	10	12	13	15	17	10	9	10	9	20
Gram Positive Bacteria:																*Gentamycin*
*Staphylococcus aureus* (RCMB010010)	NA	NA	NA	NA	NA	NA	NA	NA	NA	NA	NA	10	NA	11	8	24
*Bacillus subtilis* RCMB 015 (1) NRRL B-543	NA	NA	NA	NA	NA	NA	NA	NA	NA	NA	NA	NA	NA	NA	NA	26
Gram Negatvie Bacteria:																*Gentamycin*
*Escherichia coli* (RCMB 010052) ATCC 25955	12	13	18	NA	8	NA	10	12	NA	13	9	NA	NA	11	12	30
*Salmonella typhimurium* RCMB 006 (1) ATCC 14028	NA	NA	NA	NA	15	NA	NA	NA	NA	7	8	10	NA	NA	NA	17

The test was done using the diffusion agar technique, well diameter: 6.0 mm (100 µL was tested). NA: No activity. All sample at 5 mg/mL.

**Table 3:** The IC_50_ value of antioxidant activity for newly synthesized compounds **1**–**15**.

Compound Code	IC_50_ (μg/mL)
Tocopherol	6.78
**1**	22.8
**2**	>1000
**3**	252.7
**4**	>1000
**5**	110.3
**6**	70.5
**7**	700
**8**	-
**9**	602.5
**10**	-
**11**	508.5
**12**	242.7
**13**	218.5
**14**	3.49
**15**	4.7

## References

[1] Senderowicz A.M. (2004). Targeting cell cycle and apoptosis for the treatment of human malignancies. Curr. Opin. Cell Biol..

[2] Rashad A.E., Shamroukh A.H., El-Hashash M.A., El-Farargy A.F., Yousif N.M., Salama M.A., Mostafa A., El-Shahat M. (2012). Synthesis and Anti-Avian Influenza Virus (H5N1) Evaluation of Some Novel Nicotinonitriles and Their *N*–Acylic Nucleosides. J. Heterocycl. Chem..

[3] Tanifum E.A., Kots A.Y., Choi B.K., Murad F., Gilbertson S.R. (2009). Novel pyridopyrimidine derivatives as inhibitors of stable toxin a (STa) induced cGMP synthesis. Bioorg. Med. Chem. Lett..

[4] Rosowsky A., Mota C.E., Queener S.F. (1995). Synthesis and antifolate activity of 2, 4-diamino-5,6,7,8-tetrahydropyrido [4,3-*d*] pyrimidine analogues of trimetrexate and piritrexim. J. Heterocycl. Chem..

[5] Kotb E.R., Abbas H.A.S., Flefel E.M., Sayed H.H., Abdelwahed N.A.M. (2015). Utility of Hantzsch ester in synthesis of some 3,5-bis-dihydropyridine derivatives and studying their biological evaluation. J. Heterocycl. Chem..

[6] Flefel E.M., Abbas H.A.S., Abdel Magid R.E., Zaghary W.A. (2016). Synthesis and Cytotoxic Effect of Some Novel 1,2-Dihydropyridine-3-Carbonitrile and Nicotinonitrile Derivatives. Molecules.

[7] El-Sayed A.A., Khaireldin N.Y., El-Shahat M., El-Hefny E.A., El-Saidi M.M.T., Ali M.M., Mahmoud A.E. (2016). Antiproliferative Activity For Newly Heterofunctionalized Pyridine Analogues. Ponte.

[8] Sayed H.H., Morsy E.M., Flefel E.M. (2010). Synthesis and reactions of some novel Nicotinonitrile, thiazolotriazole, and Imidazolotriazole derivatives for Antioxidant evaluation. Synth. Commun..

[9] Sayed H.H., Flefel E.M., Abd El-Fatah A.M., El-Sofany W.I. (2010). Focus on the Synthesis and Reactions of Some New Pyridine Carbonitrile Derivatives as Antimicrobial and Antioxidant Agents. Egypt. J. Chem..

[10] Abdelhameed R.M., El-Sayed H.A., El-Shahat M., El-Sayed A.A., Darwesh O.M. (2018). Novel triazolothiadiazole and triazolothiadiazine derivatives containing pyridine moiety: Design, synthesis, bactericidal and fungicidal activities. Curr. Bioact. Compd..

[11] Abo-Ghalia M.H., Amr A.E.G.E., Abdalah M.M. (2003). Synthesis of some new (Nα-dipicolinoyl)-bis-L-leucyl-DL-norvalyl linear tetra and cyclic octa bridged peptides as new antiinflammatory agents. Zeitschrift für Naturforschung B.

[12] Sondhi S.M., Dinodia M., Kumar A. (2006). Synthesis, anti-inflammatory and analgesic activity evaluation of some amidine and hydrazone derivatives. Bioorg. Med. Chem..

[13] Komoda H., Inoue T., Node K. (2010). Anti-inflammatory properties of azelnidipine, a dihydropyridine-based calcium channel blocker. Clin. Exp. Hypertens..

[14] Márquez-Flores Y.K., Campos-Aldrete M.E., Salgado-Zamora H., Correa-Basurto J., Meléndez-Camargo M.E. (2012). Docking simulations, synthesis, and anti-inflammatory activity evaluation of 2-(*N*-alkyl) amino-3-nitroimidazo [1,2-*a*]pyridines. Med. Chem. Res..

[15] Coburn R.A., Wierzba M., Suto M.J., Solo A.J., Triggle A.M., Triggle D.J. (1988). 1, 4-Dihydropyridine antagonist activities at the calcium channel: a quantitative structure-activity relationship approach. J. Med. Chem..

[16] Lim J.T., Piazza G.A., Han E.K.H., Delohery T.M., Li H., Finn T.S., Gross P.H. (1999). Sulindac derivatives inhibit growth and induce apoptosis in human prostate cancer cell lines. Biochem. Pharmacol..

[17] Kotb E.R., El-Hashash M.A., Salama M.A., Kalf H.S., Abdel Wahed N.A. (2009). Synthesis and reactions of some novel nicotinonitrile derivatives for anticancer and antimicrobial evaluation. Acta Chim. Slov..

[18] Al-Abdullah E.S. (2011). Synthesis and anticancer activity of some novel tetralin-6-yl-pyrazoline, 2-thioxopyrimidine, 2-oxopyridine, 2-thioxo-pyridine and 2-iminopyridine derivatives. Molecules.

[19] Das K.S., Dey S.K., Maity S., Guha P., Choubey M., Bandyopadhyay U. (2008). Antiplasmodial activity of [(aryl) arylsulfanylmethyl] pyridine. Antimicrob. Agents Chemother..

[20] Patel N.B., Agravat S.N., Shaikh F.M. (2011). Synthesis and antimicrobial activity of new pyridine derivatives-I. Med. Chem. Res..

[21] Kotb E.R., Anwar M.M., Abbas H.A.S., Abd E.M., SI A. (2013). A concise synthesis and antimicrobial activity of a novel series of naphthylpyridine-3-carbonitrile compounds. Acta Pol. Pharm..

[22] Acharya B.N., Thavaselvam D., Kaushik M.P. (2008). Synthesis and antimalarial evaluation of novel pyridine quinoline hybrids. Med. Chem. Res..

[23] Mukai A., Nagai A., Inaba S., Takagi M., Shin-ya K. (2009). JBIR-54, A new 4-pyridinone derivative isolated from Penicillium daleae Zaleski fE50. J. Antibiot..

[24] Nigade G., Chavan P., Deodhar M. (2012). Synthesis and analgesic activity of new pyridine-based heterocyclic derivatives. Med. Chem. Res..

[25] Amr A.E.G.E., Sayed H.H., Abdulla M.M. (2005). Synthesis and reactions of some new substituted pyridine and pyrimidine derivatives as analgesic, anticonvulsant and antiparkinsonian agents. Arch. Pharm. Chem. Life Sci..

[26] Worachartcheewan A., Prachayasittikul S., Pingaew R., Nantasenamat C., Tantimongcolwat T., Ruchirawat S., Prachayasittikul V. (2012). Antioxidant, cytotoxicity, and QSAR study of 1-adamantylthio derivatives of 3-picoline and phenylpyridines. Med. Chem. Res..

[27] Mu J.X., Shi Y.X., Wu H.K., Sun Z.H., Yang M.Y., Liu X.H., Li B.J. (2016). Microwave assisted synthesis, antifungal activity, DFT and SAR study of 1,2,4-triazolo[4,3-*a*]pyridine derivatives containing hydrazone moieties. Chem. Cent. J..

[28] Wang Q., Zhai Z., Sun Z., Liu X., Tan C., Weng J. (2016). Synthesis, Crystal Structure and Antifungal Activity of 8-Chloro-3-((4-chlorobenzyl)-thio)[1,2,4]triazolo[4,3-*a*] pyridine. Chin. J. Struct. Chem..

[29] Zhai Z.W., Shi Y.X., Yang M.Y., Zhao W., Sun Z.H., Weng J.Q., Zhang Y.G. (2016). Microwave assisted synthesis and antifungal activity of some novel thioethers containing 1,2,4-triazolo[4, 3-*a*] pyridine moiety. Lett. Drug Des. Discov..

[30] Xu F.Z., Shao J.H., Zhu Y.Y., Liu L.W., Zhao Y.H., Shan W.L., Xue W. (2017). Synthesis, antifungal and insecticidal activity of novel[1,2,4]triazolo[4, 3-*a*] pyridine derivatives containing a sulfide substructure. Chem. Pap..

[31] Liu X.H., Xu X.Y., Tan C.X., Weng J.Q., Xin J.H., Chen J. (2015). Synthesis, crystal structure, herbicidal activities and 3D-QSAR study of some novel 1,2,4-triazolo [4,3-*a*] pyridine derivatives. Pest Manag. Sci..

[32] Guan L.P., Zhang R.P., Sun Y., Chang Y., Sui X. (2012). Synthesis and studies on the anticonvulsant activity of 5-alkoxy-[1,2,4]triazolo[4,3-*a*]pyridine derivatives. Arzneimittelforschung.

[33] Prakash O., Hussain K., Aneja D.K., Sharma C., Aneja K.R. (2011). A facile iodine (III)-mediated synthesis of 3-(3-aryl-1-phenyl-1*H*-pyrazol-4-yl)-[1,2,4]triazolo[4,3-*a*]pyridines via oxidation of 2-((3-aryl-1-phenyl-1*H*-pyrazol-4-yl)methylene)-1-(pyridin-2-yl)hydrazines and their antimicrobial evaluations. Org. Med. Chem. Lett..

[34] Sadana A.K., Mirza Y., Aneja K.R., Prakash O. (2003). Hypervalent iodine mediated synthesis of 1-aryl/hetryl-1,2,4-triazolo [4,3-*a*] pyridines and 1-aryl/hetryl 5-methyl-1,2,4-triazolo [4,3-*a*] quinolines as antibacterial agents. Eur. J. Med. Chem..

[35] Maqbool M., Manral A., Jameel E., Kumar J., Saini V., Shandilya A., Jayaram B. (2016). Development of cyanopyridine–triazine hybrids as lead multitarget anti-Alzheimer agents. Bioorg. Med. Chem..

[36] Ibrahim M.A., Abdel-Rahman R.M., Abdel-Halim A.M., Ibrahim S.S., Allimony H.A. (2009). Synthesis, chemical reactivity and fungicidal activity of pyrido [1,2-*b*][1,2,4] triazine derivatives. J. Braz. Chem. Soc..

[37] Ali T.E.S., Ibrahim M.A. (2010). Synthesis and antimicrobial activity of chromone-linked 2-pyridone fused with 1,2,4-triazoles, 1,2,4-triazines and 1,2,4-triazepines ring systems. J. Braz. Chem. Soc..

[38] Pawlak D., Pawlak K., Chabielska E., Małyszko J., Takada A., Myśliwiec M., Buczko W. (1998). A potent 5-hydroxytryptamine receptor (5-HT2A) antagonist, DV-7028, delays arterial thrombosis development in rats. Thromb. Res..

[39] Pawlak D., Adamkiewicz M., Malyszko J., Takada A., Mysliwiec M., Buczko W. (1998). Vascular and cardiac effects of DV-7028, a selective, 5-HT2-receptor antagonist in rats. J. Cardiovasc. Pharmacol..

[40] Watanabe Y., Usui H., Kobayashi S., Yoshiwara H., Shibano T., Tanaka T., Kanao M. (1992). Syntheses and 5-HT2 antagonist activity of bicyclic 1,2,4-triazol-3(2*H*)-one and 1,3,5-triazine-2,4 (3*H*)-dione derivatives. J. Med. Chem..

[41] Tanitame A., Oyamada Y., Ofuji K., Fujimoto M., Iwai N., Hiyama Y., Nagai K. (2004). Synthesis and antibacterial activity of a novel series of potent DNA gyrase inhibitors. Pyrazole derivatives. J. Med. Chem..

[42] Bekhit A.A., Abdel-Aziem T. (2004). Design, synthesis and biological evaluation of some pyrazole derivatives as anti-inflammatory-antimicrobial agents. Bioorg. Med. Chem..

[43] Bekhit A.A., Ashour H., Guemei A.A. (2005). Novel Pyrazole Derivatives as Potential Promising Anti-inflammatory Antimicrobial Agents. Arch. Pharm. Chem. Life Sci..

[44] Bondock S., Fadaly W., Metwally M.A. (2010). Synthesis and antimicrobial activity of some new thiazole, thiophene and pyrazole derivatives containing benzothiazole moiety. Eur. J. Med. Chem..

[45] Gökhan-Kelekçi N., Yabanoğlu S., Küpeli E., Salgın U., Özgen Ö., Uçar G., Bilgin A.A. (2007). A new therapeutic approach in Alzheimer disease: some novel pyrazole derivatives as dual MAO-B inhibitors and antiinflammatory analgesics. Bioorg. Med. Chem..

[46] Burguete A., Pontiki E., Hadjipavlou-Litina D., Villar R., Vicente E., Solano B., Monge A. (2007). Synthesis and anti-inflammatory/antioxidant activities of some new ring substituted 3-phenyl-1-(1, 4-di-*N*-oxide quinoxalin-2-yl)-2-propen-1-one derivatives and of their 4, 5-dihydro-(1*H*)-pyrazole analogues. Bioorg. Med. Chem Lett..

[47] Manojkumar P., Ravi T., Subbuchettiar G. (2009). Synthesis of coumarin heterocyclic derivatives with antioxidant activity and in vitro cytotoxic activity against tumour cells. Acta Pharm..

[48] Nitulescu G.M., Draghici C., Missir A.V. (2010). Synthesis of new pyrazole derivatives and their anticancer evaluation. Eur. J. Med. Chem..

[49] Lv P.C., Li H.Q., Sun J., Zhou Y., Zhu H.L. (2010). Synthesis and biological evaluation of pyrazole derivatives containing thiourea skeleton as anticancer agents. Bioorg. Med. Chem..

[50] Balbi A., Anzaldi M., Macciò C., Aiello C., Mazzei M., Gangemi R., Viale M. (2011). Synthesis and biological evaluation of novel pyrazole derivatives with anticancer activity. Eur. J. Med. Chem..

[51] Vijesh A.M., Isloor A.M., Telkar S., Arulmoli T., Fun H.K. (2013). Molecular docking studies of some new imidazole derivatives for antimicrobial properties. Arab. J. Chem..

[52] Chmara H., Andruszkiewicz R., Borowski E. (1984). Inactivation of glucosamine-6-phosphatesynthetase from Salmonella†typhimurium†LT2 SL 1027 by N-beta-fumarylcarboxyamido-l-2,3-diamino-propionic acid. Biochem. Biophys. Res. Commun..

[53] Marshall N.J., Andruszkiewicz R., Gupta S., Milewski S., Payne J.W. (2003). Structure activity relationships for a series of peptidomimetic antimicrobial prodrugs containing glutamine analogues. J. Antimicrob. Chemother..

[54] Borowski E. (2000). Novel approaches in the rational design of antifungal agents of low toxicity. Farmaco.

[55] Magdy M.Y., Mohammed A.A., Mohamed A.I. (2012). Synthesis, DNA affinity, and antimicrobial activity of 4-substituted phenyl-2,2′-bichalcophenes and aza-analogues. Med. Chem. Res..

[56] Hassan A.E., Ahmed H.M., Abd El-Fattah Z.H., Rajab A., El Sayed H.E. (2011). Synthesis, antitumor and antimicrobial activities of 4-(4-chlorophenyl)-3-cyano-2-(β-O-glycosyloxy)-6-(thien-2-yl)- nicotinonitrile. Eur. J. Med. Chem..

[57] Umesh D.P., Ajay P.N., Pramod S.N., Pramod P.M. (2012). Synthesis, antimicrobial and antifungal activity evaluation of 2,6-diaryl-4-SEC-aminonicotinonitriles and 4-sec-amino-6-aryl-2-(pyridin-2-yl)pyridine-3-carbonitriles i.e., Bipyridines and their docking study. J. Pharm. Res..

[58] Rashad A.E., Shamroukh A.H., Yousif N.M., Salama M.A., Ali M.M., Mahmoud A.E., El-Shahat M. (2012). New Pyrimidinone and Fused Pyrimidinone Derivatives as Potential Anticancer Chemotherapeutics. Arch. Pharm. Chem. Life Sci..

[59] Shamroukh A.H., El-Shahat M., Drabowicz J., Ali M.M., Rashad A.E. (2013). Anticancer evaluation of some newly synthesized N-nicotinonitrile derivative. Eur. J. Med. Chem..

[60] Flefel E.M., Tantawy W.A., El-Sofany W.I., El-Shahat M., El-Sayed A.A., Abd-Elshafy D.N. (2017). Synthesis of Some New Pyridazine Derivatives for Anti-HAV Evaluation. Molecules.

[61] El-Sayed A.A., Khalil A.M., El-Shahat M., Khaireldin N.Y., Rabie S.T. (2016). Antimicrobial activity of PVC-pyrazolone-silver nanocomposites. J. Macromol. Sci. Part A Pure Appl. Chem..

[62] El-Shahat M. (2007). Studies on the Synthesis and Chemical Reactions of Some Mixed and Non-Mixed Heterocyclic Compounds of Expected Biological Activity. Master’s Thesis.

[63] Hindler J.A., Howard B.J., Keiser J.F. (1994). Antimicrobial agents and antimicrobial susceptibility testing. Clinical and Pathogenic Microbiology.

[64] Yen G.C., Duh P.D. (1994). Scavenging effect of methanolic extracts of peanut hulls on free-radical and active-oxygen species. J. Agric. Food Chem..

[65] Morris G.M., Goodsell D.S., Halliday R.S., Huey R., Hart W.E., Belew K.R., Olson A.J. (1998). Automated docking using a Lamarckian genetic algorithm and empirical binding free energy function. J. Comput. Chem..

[66] Noel M.O., Michael B., Craig A.J., Chris M., Tim V., Geoffrey R.H. (2011). Open Babel: An open chemical toolbox. J. Cheminform..

[67] Mouilleron S., Badet-Denisot M.A., Golinelli-Pimpaneau B. (2008). Ordering of C-terminal loop and glutaminase domains of glucosamine-6-phosphate synthase promotes sugar ring opening and formation of the ammonia channel. J. Mol. Biol..

[68] Schüttelkopf A.W., Aalten D.M. (2004). PRODRG: A tool for high-throughput crystallography of protein-ligand complexes. Acta Cryst..

